# Core-shell microneedles Co-loaded with simvastatin and doxycycline for combined anti-infection and osteogenesis in diabetic periodontitis

**DOI:** 10.1016/j.mtbio.2026.103266

**Published:** 2026-05-21

**Authors:** Shengdan Zhang, Wen Zhang, You Wang, Miao Yin, Shuai Lu, Wei Li, Bo Cheng

**Affiliations:** a Department of Stomatology, Zhongnan Hospital of Wuhan University, Wuhan University, Wuhan, 430071, PR China; b School of Pharmaceutical Sciences, Wuhan University, Wuhan, 430071, PR China; c Department of Orthopedic Trauma, Beijing Jishuitan Hospital, Capital Medical University, Beijing, PR China; d TaiKang Center for Life and Medical Sciences, Wuhan University, Wuhan, 430071, PR China

## Abstract

Diabetic periodontitis remains a formidable clinical challenge characterized by persistent infection, oxidative stress, and impaired alveolar bone regeneration. Here, we have developed an engineered core-shell microneedle (MN) patch that enables the staged release of doxycycline and simvastatin to achieve coordinated antibacterial and osteogenic effects within periodontal lesions. The outer polyvinyl alcohol (PVA) shell rapidly dissolves upon insertion into gingival tissue, enabling burst release of doxycycline for immediate bacterial eradication. Subsequently, the polyvinylpyrrolidone (PVP) core, loaded with simvastatin-encapsulated polydopamine nanoparticles (Sim@PDA NPs), provides sustained release, leveraging the intrinsic reactive oxygen species (ROS)-scavenging capacity of PDA to mitigate local oxidative stress and promote osteogenic differentiation. A silk fibroin backing layer ensures long-term doxycycline release, preventing bacterial recolonization and maintaining a regenerative microenvironment. *In vitro*, Doxy/SIM-MNs exhibited potent antibacterial and antibiofilm activity against *Porphyromonas gingivalis* and *Staphylococcus aureus*, excellent cytocompatibility, and robust induction of alkaline phosphatase activity, mineralized nodule formation, and osteogenic gene expression in mesenchymal stem cells. In a rat model of diabetic periodontitis, Doxy/SIM-MNs markedly reduced inflammatory destruction, enhanced alveolar bone regeneration, and reestablished periodontal homeostasis by relieving negative regulation of Wnt signaling while suppressing NF-κB signaling and apoptosis. This hierarchically staged microneedle platform offers a clinically translatable strategy for the integrated management of infection, inflammation, and bone regeneration in diabetes-associated periodontitis and other chronic inflammatory bone diseases.

## Highlights

• A core-shell microneedle (MN) patch is designed for spatiotemporally coordinated drug delivery in diabetic periodontitis.
• The MN enables staged release: burst antibacterial release followed by sustained antibacterial inhibition and osteogenesis.
• The strategy outperforms conventional treatment by coordinating infection control with tissue regeneration.
• This platform offers a translatable approach for managing complex inflammatory bone diseases.

## Graphical abstract

**Figure:**
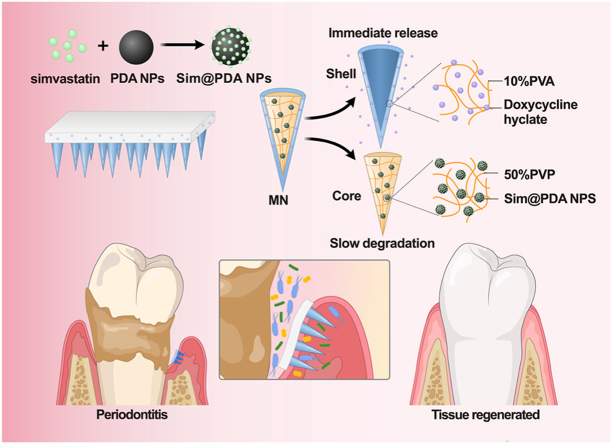

## 1 Introduction

Periodontitis is a chronic inflammatory condition affecting the periodontal supporting structures, initiated by microbial infections derived from dental plaque. This pathological process promotes the development of deep periodontal pockets, progressive attachment loss, and alveolar bone resorption [1,2]. If untreated, the disease can advance to tooth mobility and eventual tooth loss, significantly impairing overall oral function and health [3,4]. Due to its remarkably high prevalence, periodontitis ranks among the most common oral diseases in humans and remains a significant public health challenge worldwide [5]. Studies conducted between 2011 and 2020 indicate that the combined prevalence of periodontitis reaches nearly 62% in dentate adults. Specifically, moderate-to-severe cases account for an estimated 53.2%, with severe periodontitis affecting 23.6% of this population [6,7]. Periodontitis is also considered a global public health problem, not only affecting periodontal health but also having implications for the patient's general health and wellbeing [8,9]. Growing clinical and epidemiological evidence demonstrates strong associations between periodontitis and a wide spectrum of systemic diseases. Including cardiovascular disorders, type 2 diabetes, respiratory infections, adverse pregnancy outcomes, neurodegenerative disorders, and various cancers, highlighting the systemic significance of oral health [[10], [11], [12], [13], [14], [15], [16]]. Diabetes has been unequivocally established as a major risk factor for periodontitis [[17], [18], [19]]. Epidemiological evidence indicates that individuals with diabetes face an approximately threefold increased risk of developing periodontitis compared to those without diabetes [[20], [21], [22]]. Extensive clinical evidence supports a bidirectional relationship between diabetes mellitus and periodontitis-diabetes increases the risk and severity of periodontitis, while advanced periodontitis impairs glycemic control and accelerates diabetic complications [[23], [24], [25]].

Currently, standard clinical treatments for periodontitis, including supragingival scaling, subgingival curettage, and root planning, can slow its progression but are unable to fully restore the damaged soft and hard periodontal tissues [[26], [27], [28], [29]]. The most desirable clinical outcome in periodontitis treatment is the regeneration of periodontal tissues, particularly of alveolar bone regeneration. However, in diabetes, hyperglycemia aggravates periodontal inflammation via excessive production of reactive oxygen species (ROS), advanced glycation end products (AGEs), and other proinflammatory mediators [[30], [31], [32]]. The prolonged inflammatory microenvironment profoundly impairs the regenerative capacity of periodontal cells, resulting in irreversible tissue destruction [33,34]. Therefore, patients with diabetes, especially those with poor glycemic control, frequently develop severe periodontitis with multiple abscesses, requiring combined mechanical and antibiotic therapy. While local antibiotic delivery offers better efficacy and fewer systemic side effects than oral administration, it struggles to sustain effective drug concentrations in the periodontal pocket due to clearance from saliva and oral functions. Accordingly, the development of innovative therapeutic strategies that integrate antibacterial activity with promotion of periodontal tissue regeneration under diabetic conditions holds profound scientific and clinical significance.

The limitations of conventional monotherapies have spurred interest in combination strategies that simultaneously target multiple pathogenic pathways. In this study, we developed a dissolving microneedle (MN) patch co-loaded with doxycycline (Doxy) and simvastatin (SIM) based on a compelling complementary rationale. Doxycycline, a semi-synthetic tetracycline antibiotic, is a cornerstone in periodontitis management [35]. It exhibits broad-spectrum antibacterial activity and demonstrates strong inhibitory effects against key periodontal pathogens such as *Porphyromonas gingivalis* (*P. gingivalis*), *Aggregatibacter actinomycetemcomitans* (*A. actinomycetemcomitans*), and *Prevotella intermedia* (*P. intermedia*). Beyond its direct antimicrobial action, its therapeutic efficacy at subantimicrobial doses is primarily attributed to its potent inhibition of matrix metalloproteinases (MMPs) and its modulation of the host immune-inflammatory response, effectively mitigating soft tissue degradation and alveolar bone loss [36]. However, systemic administration of doxycycline can lead to adverse effects such as gastrointestinal disturbances and disruption of the normal microbiota [37]. Therefore, local drug delivery is considered an ideal approach for periodontal therapy to maximize efficacy while minimizing systemic side effects.

Simvastatin, originally developed as a lipid‐lowering drug for cardiovascular disease [38], has been increasingly reported to exert pleiotropic effects, such as anti‐inflammatory, pro-angiogenic, and osteogenic activities. At concentrations ranging from 0.2 to 1 μM, simvastatin has been shown to dose‐dependently enhance osteogenic differentiation of mouse bone marrow mesenchymal stem cells [39], bone marrow cells [40], and MC3T3‐E1 cells [41], while significantly upregulating osteogenic gene expression including *BMP2*, *ALP*, and *OCN* [42,43]. In primary alveolar osteoblasts and periodontal ligament cells, simvastatin increased osteogenic marker expression, and local delivery of simvastatin into periodontal pockets elevated alveolar crest height and prevented bone loss in ovariectomized (OVX) rats with experimental periodontitis [44]. As a lipophilic anionic compound, simvastatin administered orally undergoes substantial hepatic and renal clearance, necessitating higher systemic doses to achieve therapeutic levels at periodontal defects—thereby increasing the risk of adverse events such as hepatic or renal toxicity and exacerbated inflammation. In contrast, local delivery not only enhances osteogenesis at the defect site but also reduces off‐target adverse effects. Polydopamine Nanoparticles (PDA NPs) are particularly suitable as drug carriers due to their intrinsic antioxidant activity [45], their robust entry to different cell types [46], biocompatibility [47], ease of surface modification with biomolecules [48] and high drug-loading capacity [49]. On this basis, we developed PDA-based nanocarriers for the co-delivery of simvastatin, thereby providing localized osteogenic stimulation in combination with inherent antioxidant protection within periodontal tissues.

It should be emphasized that conventional physical encapsulation of therapeutic agents frequently results in rapid burst release, thereby compromising drug bioavailability and limiting clinical efficacy [50,51]. These shortcomings underscore the urgent need for advanced delivery systems capable of co-loading anti-inflammatory and osteoinductive agents while ensuring controlled and sustained release within periodontal lesions. In this context, MN patches have emerged as a promising platform, offering the capability for staged or sequential release of multiple therapeutic cargos to overcome the inherent limitations of traditional approaches [[52], [53], [54], [55]]. To support this notion, we report the design of core-shell structured MN patches for synchronized intra‐pocket delivery of antibacterial and osteoinductive agents. The core-shell MNs were designed for a sequential therapeutic strategy: to initially eradicate pathogenic microorganisms, followed by modulation of the periodontal microenvironment to facilitate bone regeneration. (Fig. 1). Upon insertion into deep periodontal pockets, doxycycline embedded in the rapidly dissolving Poly (vinyl alcohol) (PVA) outer layer was released within 20 min, efficiently eliminating bacterial biofilms. Subsequently, swelling of the PVP inner core enabled sustained release of simvastatin loaded in PDA NPs, thereby promoting alveolar bone regeneration. Meanwhile, doxycycline incorporated in the silk fibroin backing layer was gradually released for over two weeks, effectively preventing bacterial recolonization, reducing inflammation relapses, and establishing a favorable microenvironment for tissue repair. Collectively, MN patches designed for microenvironment‐responsive and controllable drug release highlights a transformative strategy for local drug delivery in periodontitis.

**Fig. 1:**
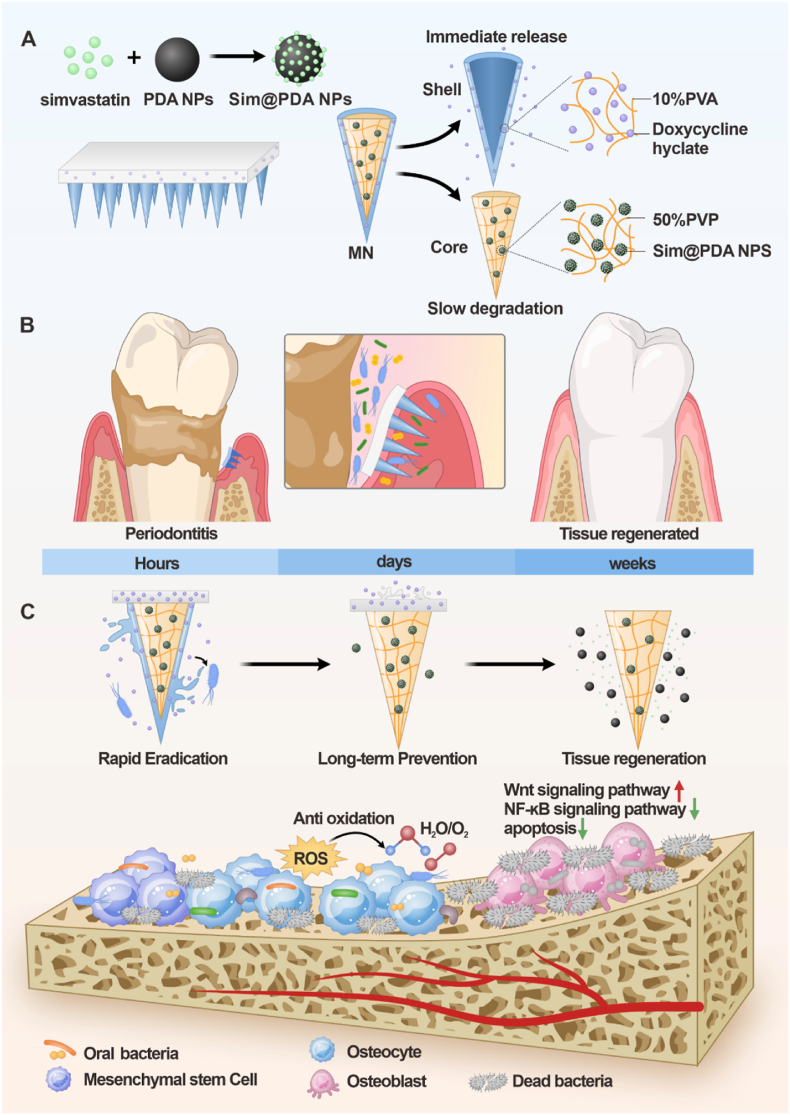
Schematic illustration of the Doxy/SIM-MNs for Periodontitis with Diabetes Mellitus treatment.

## 2 Materials and methods

### 2.1 Reagents

All reagents and materials used in this study were sourced from commercial suppliers and validated for experimental use. Dopamine hydrochloride, Doxycycline hydrochloride, were purchased from Aladdin Reagent Co. (Shanghai, China). Ethanol, Sodium hydroxide (NaOH), hydrochloric acid (HCl), Disodium ethylene diamine tetraacetate (EDTA), lithium bromide (LiBr) and Sodium carbonate (Na_2_CO_3_) were purchased from Sinopharm Chemical Reagent Co. (Shanghai, China). Polyvinylpyrrolidone (PVP, K30) was purchased from Vokai Biotechnology Co. (Beijing, China). Polyvinyl alcohol (PVA, Mw 146,000∼186,000) was purchased from Sigma-Aldrich (St. Louis, MO, USA). *B. mori* cocoons were purchased from cocoon company (Yuhang, China). Fluorescein isothiocyanate (FITC), vitamin K1, L-Cysteine were purchased from Macklin Biochemical Technology Co. (Shanghai, China). TSB nutrient broth and TSB nutrient agar were purchased from Hopebio Biotechnology Co. (Qingdao, China). Yeast extract was purchased from Oxoid Ltd. (Hampshire, UK). LIVE/DEAD bacterial viability kits were purchased from Thermo Fisher Scientific (Waltham, USA). High-glucose Dulbecco's modified Eagle medium (DMEM), fetal bovine serum (FBS), and penicillin-streptomycin antibiotics were obtained from Procell Life Science & Technology Co. (Wuhan, China). Calcein- AM/PI cell viability/cytotoxicity assay kit, Cell Counting Kit-8 (CCK-8), Alizarin Red S Staining Kit for Osteogenesis and ALP detection kit were purchased from Beyotime Biotechnology Co. (Shanghai, China). Runx2 rabbit pAb and Osteocalcin rabbit mAb for Western blot were obtained from Abclonal Technology Co. (Wuhan, China). All chemical reagents are purified by any means. *Porphyromonas gingivalis* strain ATCC 33277 and *Staphylococcus aureus* were obtained from the Shanghai Microbial Culture Collection Center (Shanghai, China). Murine C3H10T1/2 cells were obtained from Procell Life Science & Technology Co (Wuhan, China), and were cultured using DMEM medium containing 10% FBS and 1% penicillin-streptomycin at 37 °C with 5% CO_2_. Male Sprague-Dawley (SD) rats were purchased from Hubei Bennt Biological Technology Co. (Wuhan, China). The experimental protocol was approved by the Institutional Animal Care and Use Committee of Wuhan University (WP20230748).

### 2.2 Synthesis and evaluation of Sim@PDA NPs

#### 2.2.1 Chemical synthesis of Sim@PDA NPs

PDA NPs were prepared by the oxidative self-polymerization of DA-HCl in an alkaline aqueous medium mentioned in literature with a small modification [49,56]. In brief, 100 mg dopamine hydrochloride was dissolved in 100 mL deionized water under magnetic stirring until the solution transitioned from brownish-yellow to pale yellow. The mixture was heated to 50 °C in an oil bath, followed by dropwise addition of 1 mL 1M NaOH under aerobic conditions with 24 h dark stirring. The reaction was terminated when the solution turned black. The resulting product was centrifuged at 11,000 rpm for 15 min, washed 3 times with deionized water, lyophilized, and stored at −20 °C. Subsequently, 150 mg of simvastatin was dissolved in 50 mL of anhydrous ethanol and mixed with 100 mg of lyophilized PDA NPs under stirring for 12 h to obtain Sim@PDA NPs. Unbound drug was removed by centrifugation (11,000 rpm, 15 min) followed by ethanol washing until the supernatant became clear. The drug loading efficiency (DLE) and encapsulation efficiency (EE) were determined by measuring the concentration of unbound simvastatin in the combined supernatant and washing solutions using ultraviolet-visible (UV-Vis) spectrophotometry. DLE and EE were calculated using the following equations: DLE (%) = (Mass of drug in NPs/Mass of NPs) × 100; EE (%) = (Mass of drug in NPs/Mass of drug fed initially) × 100. The Sim@PDA NPs was then stored at 4 °C.

#### 2.2.2 Physicochemical, morphological characterization and drug release study *in vitro*

The polydispersity index (PDI) of the obtained Sim@PDA NPs was examined by a dynamic light scattering analyzer (Zeta-Sizer, UK). The morphology of the synthesized Sim@PDA NPs and PDA NPs were conducted via scanning electron microscopy (SEM, Tescan, Chezch Republic) and transmission electron microscopy (TEM, Hitachi, HT7700). UV-vis spectra were recorded using a UV-vis spectrophotometer (UV-vis) (Shimadzu, UV-2600). The infrared spectrum of nanoparticles was acquired using an attenuated total reflectance Fourier transform infrared spectroscopy (ATR/FT-IR) system (Thermo Fisher, Nicolet iS50)). The surface chemical composition and states were analyzed by X-ray photoelectron spectroscopy (XPS) on a Kratos AXIS Ultra DLD spectrometer using a monochromatic Al Kα X-ray source. The drug (Simvastatin) release from Sim@PDA NPs was studied separately using a dialysis method. Briefly, Sim@PDA NPs solutions (2 mg/mL, 1 mL each) were added to disposable dialysis bags (MWCO: 3500 Da, Thermo Scientific). The dialysis bags were then immersed in 10 mL of in the PBS (release medium, pH 7.4) at 37 °C. Three independent replicates were used for each sample. Two milliliters of release medium were collected for analysis at different time intervals and replaced with an equivalent volume of fresh solutions at 37 °C. The release medium was analyzed by UV-vis spectrophotometer. The wavelength is set at 238 nm.

#### 2.2.3 Cytocompatibility evaluation of CCK-8 assay

The cytotoxicity of Sim@PDA NPs at various concentrations (0, 6.125, 12.5, 25, 50, 100 and 200 μg/mL) was evaluated using the CCK-8 kit, following standard operational guidelines. C3H10T1/2 cells were seeded into 96-well plates at a density of 5 × 10^3^ cells per well and cultured for 24 h in a humidified incubator at 37 °C with 5% CO_2_. Subsequently, the cells were treated with different concentrations of Sim@PDA NPs and incubated for another 24 h. Thereafter, 10 μL of CCK-8 solution was added to each well, followed by incubation for 2 h. Finally, the absorbance of each well at 450 nm was measured using a microplate reader.

#### 2.2.4 ROS scavenging assay of Sim@PDA NPs

**Determination of SOD Activity:**In this study, the activity of superoxide dismutase (SOD) was determined using the pyrogallol autoxidation method [57,58]. A fresh 7.5 mM pyrogallol stock solution was prepared by dissolving pyrogallol in 10 mM HCl. A 50 mM Tris-HCl buffer (pH 8.2) was accurately prepared. The NPs to be tested were dispersed in deionized water at a concentration of 4 mg/ml. A 3 mL reaction system was established as follows: 2.97 mL Tris-HCl buffer, 10 μL HCl solution and 20 μL pyrogallol stock solution in control group; 2.97 mL Tris-HCl buffer, 10 μL NPs suspension and 20 μL pyrogallol stock solution in experimental group. The reaction was immediately monitored using a UV-vis spectrophotometer. Scanning was performed within the wavelength range of 200-800 nm, and the absorbance at 320 nm was recorded. The inhibition rate of the NPs on the autoxidation of pyrogallol was calculated accordingly.

**Determination of DPPH Scavenging Rate:** 0.1 mM, 4 mL of DPPH solution was mixed with Sim@PDA NPs or ascorbic acid. The volume ratio of the sample to be tested and the DPPH solution was 1:3. Scavenging activity was assessed by monitoring the decrease in absorbance at 516 nm after 30 min in the dark. The DPPH radical scavenging activity was calculated as I = [1 − (Ai − Aj)/Ac] 100%, where Ac is the absorbance of DPPH in solution without sample, Ai is the mixture of sample and DPPH solution, and Aj is the absorbance of sample mixed with 95% ethanol.

**Measurement of Hydroxyl Radical Clearance Rate:** To produce 2,3-dihydroxybenzoic acid and 2,5-dihydroxybenzoic acid, salicylic acid was employed to capture hydroxyl radicals (·OH) generated via the Fenton reaction, with detection carried out by spectrophotometry [59,60]. The reaction was initiated by adding 0.10 mL of 0.03% H_2_O_2_ to a mixture containing 2.00 mL of FeSO_4_ solution (1.8 mmol/L), 1.50 mL of diluted salicylic acid-ethanol solution (1.8 mmol/L), and 1.00 mL of nanoparticle suspension (100 μg/mL) or distilled water (for control). After thorough mixing, the system was incubated at 37 °C for 30 min. The absorbance was measured at 510 nm. The radical scavenging rate was calculated as: Scavenging rate (%)=(A_0_−A_S_)/A_0_×100%, where A_0_ and A_S_ represent the absorbance of the control and the sample, respectively.

**In Vitro ROS Scavenging Assay:** To assess the ability of Sim@PDA NPs to reduce oxidative stress in an inflammatory environment, intracellular ROS levels were measured using the DCFH-DA fluorescence probe. C3H10T1/2 cells were treated with nanoparticles for 1 day and then exposed to 100 μM H_2_O_2_ for 30 min. The cells were counterstained with 10 μm DCFH-DA in DMEM and incubated at 37 °C with 5% CO_2_. Control cells were not treated with either the material or H_2_O_2_; the H_2_O_2_ group was treated with H_2_O_2_ alone without Sim@PDA NPs. The fluorescence intensity was analyzed by flow cytometry.

### 2.3 Fabrication of core-shell MN patch

A two-step casting process was used to fabricate the MN patch with a core-shell structure as previously described. Briefly, PVA (14.5 kD) was dissolved in DI water to prepare a 10% (w/v) solution. Then, 60 mg of doxycycline hydrochloride was dissolved in 2 mL of the solution. After that, 50 μL of the mixture was casted into the PDMS mold, followed by centrifugation at 4200 rpm for 20 min at 25 °C to form the shell of Doxy/SIM-MN patch. The inner core of the MN structure was fabricated using a similar method. Specifically, a total of 200 mg of Sim@PDA NPs were resuspended in 10 mL of 50% PVP-K30 aqueous solution. Then, 100 μL of the solution was applied onto the PDMS mold, followed by centrifugation (4200 rpm, 5 min, 4 °C). This process was repeated twice. To prepare the backing layer of the MN patch, a 10% silk fibroin solution was first prepared from silkworm cocoons according to previously reported methods and stored at −20 °C [61]. Then, 2 mL of the 10% silk fibroin solution was mixed with 25 mg of doxycycline hydrochloride until complete dissolution was achieved. During the dissolution process, gentle agitation was used to prevent premature gelation and precipitation of the silk fibroin. A volume of 100 μL of 10% silk fibroin solution was added to the mold surface at −0.1 MPa vacuum for 2 h to form the backing layer. The mold was then placed in a desiccator at room temperature for 48 h for complete drying. Thereafter, the patch was carefully peeled from the mold and stored in a desiccator until use. The morphology of the MN patch was observed using scanning electron microscopy (SEM, Tescan, Tescan VEGA Compact, The Czech Republic), and the mechanic strength was measured by a mechanical tester (Mark-10, ESM303, Mark-10 Corporation, New York, USA). To investigate the penetration capability, the MN patch was applied to porcine gingiva *ex vivo*. After 2 min, the MN patch was removed, and the porcine gingiva was examined under a microscope. In addition, the MNs-applied skin was cut into 10 μm-thick frozen sections to evaluate penetration and microcavity formation.

### 2.4 Biocompatibility evaluation

The biocompatibility of the microneedle (MN) patches was assessed using a Calcein-AM/Propidium Iodide (Ca-AM/PI) live/dead cell double staining kit. Briefly, C3H10T1/2 cells in the logarithmic growth phase were seeded into 6-well plates. To prepare the MN extracts, sterile blank or drug-loaded patches were incubated in DMEM supplemented with 10% fetal bovine serum (10 mL per patch) at 37 °C until complete dissolution, followed by sterile filtration through a 0.22 μm membrane to obtain 100% stock solutions. When cells reached approximately 65% confluence, the culture medium was replaced with fresh DMEM containing the MN extracts at specified concentrations. After 24 h of incubation, cells were washed three times with PBS, stained with 500 μL of Ca-AM/PI working solution for 30 min, and then imaged using an inverted fluorescence microscope.

### 2.5 Antimicrobial assay

#### 2.5.1 Determination of the inhibition zone

To examine the inhibition zone of the MN patches, 20 μL bacterial suspension was inoculated into BHI blood agar plates and randomly divided into six groups, Control group (without treatment), Blank group (core-shell MN patch without loading drug), Doxy group (core-shell MN patch with only loading doxycycline), SIM group (core-shell MN patch with only loading simvastatin), Doxy/SIM group (core-shell MN patch with loading doxycycline and simvastatin), Gel group (Minocycline Hydrochloride Ointment, Perio). After the culture medium was incubated for 7 days, the diameter of the inhibition zone was measured.

#### 2.5.2 Bacterial morphology observation

To evaluate bacterial morphological changes following treatment with drug-loaded MN patches, *P. gingivalis* and *S. aureus* were selected as model strains. Taking *P. gingivalis* as an example, 100 μL of bacterial suspension (1 × 10^6^ CFU mL^−1^) was added to 2 mL TBS medium containing extracts from different microneedle groups and incubated anaerobically at 37 °C for 8 h. After centrifugation (4500 rpm, 5 min) and PBS washing, bacterial pellets were subjected to SYTO-9/PI staining, and fluorescence was visualized using FITC and Cy3 channels on an inverted fluorescence microscope. In parallel, bacterial pellets were fixed with 2.5% glutaraldehyde at 4 °C for 12 h, dehydrated through graded ethanol, and dispersed in absolute ethanol. A 20 μL aliquot was placed onto silicon wafers, and bacterial ultrastructure was examined by scanning electron microscopy (SEM).

#### 2.5.3 Antibiofilm activity of Doxy/SIM MN patches

To evaluate the inhibitory effect of Doxy/SIM MN patches on biofilm formation by *P. gingivalis* and *S. aureus* respectively, with *P. gingivalis* serving as an example, the following experimental procedure was conducted. 2 mL of bacterial suspension along with the microneedle extract were introduced into a confocal dish and incubated under strict anaerobic conditions at 37 °C for 5 days. After incubation, the supernatant was carefully removed, and the biofilm was gently washed three times with PBS. The samples were then stained with crystal violet for 15 min. Following staining, the dye bound to the biofilm was dissolved using absolute ethanol, and the absorbance of the resulting solution was measured at 570 nm. In parallel, additional biofilms were stained with SYTO-9 and visualized using laser scanning confocal microscopy.

### 2.6 Osteogenic differentiation

C3H10T1/2 cells were seeded into 6-well plates at a density of 1 × 10^4^ cells per well. When cell confluence reached approximately 50%, the culture medium was replaced with osteogenic induction medium that had been pre-conditioned by incubation with the respective MN patches. Each 50 mL of osteogenic induction medium was supplemented with 500 μL of sodium β-glycerophosphate, 250 μL of vitamin C, and 5 μL of dexamethasone. On days 7 and 14 of induction, alkaline phosphatase (ALP) staining was performed using a commercial staining kit, and images were captured under an inverted microscope. Similarly, Alizarin Red S staining was conducted on days 14 and 21 to evaluate mineralized nodule formation. After 7 days of osteogenic induction, the expression of osteogenesis-related genes (*RUNX2*, *ALP*, *COL1*, and *OCN*) was quantified using quantitative real-time PCR (RT-qPCR). The primer sequences used are listed in Table S1. In addition, immunofluorescence staining of RUNX2 (ABclonal, A2851) and OCN (ABclonal, A20800) were followed the protocol outlined in the commercial antibody kit. Briefly, on days 14 and 21, the C3H10T1/2 cells under indicated treatments were harvested and washed with PBS three times. Followed by fixing in 4% paraformaldehyde (PFA) for 15 min and washed with PBS three times. Then, 4% PFA fixed cells were permeabilized with 0.5% saponin in PBS for 5 min on ice and washed with PBS three times. Then, the cells were blocked in 1% BSA containing PBS (v/v) for 1 h and stained in blocking buffer with primary antibody overnight at 4 °C. To visualize the primary antibodies, the cells were exposed to species-specific secondary antibodies conjugated to either Alexa Fluor 594 or Alexa Fluor 488 for 1 h at 4 °C. Finally, the cells were plated on slides and stained with In Situ Microplate Nuclear Stain and AntiFade (Sigma, Cat# DUO82064-1KIT), and the coverslips were mounted on slides. Images were acquired on an Olympus FV1000 fluorescence microscope. Additionally, immunoblot assays were performed to evaluate the protein expression levels of RUNX2 and OCN according to the manufacturer's instruction. Briefly, proteins were extracted and dictated to immunoblots. The RUNX2 and OCN protein level was quantified by ImageJ software.

### 2.7 Therapeutic effect of microneedles on a rat model with diabetic periodontitis

#### 2.7.1 Establishment of a diabetic periodontitis model in rats

All animal experiments were approved by the Institutional Animal Care and Use Committee of Wuhan University (WP20230748). Sprague-Dawley rats were fed a high-fat diet for two weeks to induce insulin resistance. Specifically, the animals received 10% (w/v) glucose in drinking water and were fed a high-fat diet (HFD) purchased from Wuhan Shulaibao Biological Technology Co., Ltd. (product No. SL0012), which provides 20% of calories from protein, 35% from carbohydrates, and 45% from fat. After 12 h of fasting, the rats received an intraperitoneal injection of streptozotocin (STZ) solution at a dosage of 35 mg/kg body weight. The STZ solution was prepared fresh immediately before use. On days 3, 5, 7, 10, and 14 after STZ injection, random blood glucose levels were measured via the tail vein. Rats with blood glucose levels below 16.7 mmol/L were excluded from the study. After 2 days post-STZ injection, periodontitis was induced as follows: rats were anesthetized by intraperitoneal injection of pentobarbital sodium, placed in a supine position, and a mouth opener was used to retract the incisors for intraoral access. A 0.2 mm diameter ligature wire was passed mesially and distally around the first molar, inserted into the gingival sulcus, and ligated firmly. Care was taken to avoid injury to the oral mucosa during the procedure. The ligature was checked every three days and replaced if lost.

#### 2.7.2 Treatment with microneedles on a rat model with diabetic periodontitis

A total of thirty-six healthy male Sprague-Dawley rats, 8-10 week old, weighing 180-200 g were randomly divided into six experimental groups (n = 6 per group): Control (no treatment), Blank-MNs (blank MN patches without drug loading), Doxy-MNs (MN patches with doxycycline-loaded shell), SIM-MNs (MN patches with simvastatin-loaded core only), Doxy/SIM-MNs (MN patches co-loaded with doxycycline and simvastatin), and Gel (Periocline® ointment, minocycline hydrochloride gel). After 14 days of ligature placement, the ligatures were removed. The rats then received topical applications of the corresponding MN patches (one patch per rat, applied into the periodontal pocket of the ligated molar) or gel once per week, according to their group assignments. Following two weeks of treatment, all rats were euthanized by CO_2_ inhalation. Bilateral maxillae were harvested for subsequent analysis. All specimens were excised by the same investigator to ensure consistency.

#### 2.7.3 Micro-CT analysis of alveolar bone

At the end of the experimental period, mouse maxillae were harvested and fixed in 4% paraformaldehyde for 48 h. To evaluate alveolar bone resorption, the specimens were scanned using a high-resolution microcomputed tomography (Micro-CT) system (SkyScan 1276, Bruker, Germany). The scanning parameters were set as follows: an X-ray voltage of 85 kV, a current of 200 μA, and an exposure time of 384 ms. A 1 mm aluminum filter was utilized to minimize beam hardening. The images were acquired at a magnificent spatial resolution with image pixel size 6.5 μm. The raw projection images were reconstructed using NRecon software (version 1.7.4.2, Bruker) with consistent settings for smoothing, ring artifact reduction, and pith error correction. For each specimen, the 3D orientation was standardized using DataViewer software (version 1.5.6.2, Bruker); the occlusal plane was aligned horizontally, and the sagittal plane was parallel to the midline of the palate to ensure consistency across all samples. Quantitative analysis was performed using CTAn software (version 1.8.18.0, Bruker). The region of interest (ROI) for the study was selected as the alveolar bone around the first molar in each specimen. The anterior border of the ROI was set at the mesial aspect of the mesial root of the first molar, and the posterior border at the mesial aspect of the mesial root of the second molar. The superior border was defined as the crest of the palatal alveolar bone, and the inferior border extended just below the apices of the mesial and distal roots of the first molar. Within this volume, the roots of the first molar were manually excluded by adjusting the contour of the selection mask to ensure the roots remained outside the final bone analysis volume. After this refinement, the following bone morphometric parameters were calculated to assess periodontal bone changes: Bone Volume/Total Volume (BV/TV), Trabecular Thickness (Tb.Th, mm), Trabecular Number (Tb.N, 1/mm), and Trabecular Separation (Tb.Sp, mm). The 3D reconstructed volume of each sample was visualized and analyzed using CTvox software (version 1.8.18.0,Bruker). The volume was first oriented so that the occlusal plane was horizontally aligned and the sagittal plane was set vertically to ensure consistent spatial normalization across all specimens. In this standardized orientation, the distance from the cementoenamel junction (CEJ) to the alveolar bone crest (ABC) was measured at both the mesial and distal aspects of the first molar. Three-dimensional visualization and representative images were generated using the same software.

#### 2.7.4 Histological analysis

After Micro-CT analysis, the extracted maxillae were submerged in paraformaldehyde for 48 h and subsequently decalcified using 10% ethylenediamine tetraacetic acid (EDTA) for a period of 3 weeks. Sections 8 μm-thick were prepared for subsequent staining with hematoxylin and eosin (Beyotime Biotech) to examine histological alterations. Images were acquired using an Aperio VERSA 8 (Leica) at 20×magnification.Tartrate-resistant acid phosphatase (TRAP) staining was used to assess the number of osteoclasts, 8 μm-thick sections were staining with a TRAP staining kit (Beyotime Biotech) according to the manufacturer's instructions. The numbers of TRAP + cells were quantified in five randomly selected fields of view within the furcation area or the mesial/distal alveolar crest of the first molar on each section. Images were acquired using an Aperio VERSA 8 (Leica) at 20×magnification. Quantification was performed using ImageJ v.1.52a (Bethesda). Immunohistochemical (IHC) staining for Osteocalcin (OCN) using standard protocols. Briefly, 8 μm-thick slides were deparaffinized in xylene, and rehydrated in 100%, 95% and 75% ethanol for 5 min. Antigen retrieval was performed by heating slides in a microwave for 30 min in sodium citrate buffer (pH 6.0). The sections were cooled down naturally to room temperature and quenched in 3% hydrogen peroxide to block endogenous peroxidase activity. The OCN primary antibody (Abclonal, Cat: A20800) was diluted in PBS containing 1% BSA (1:500) was incubated at 4 °C overnight followed by Maixin-Bio Detection Kit peroxidase/diaminobenzidine (DAB) rabbit/mouse (Kit-9710, DAB-0031; Maixi-Bio, Fuzhou) according to the manufacturer's instructions. Subsequently, sections were counterstained with hematoxylin (Beyotime Biotech) for 1 min and coverslipped. Images were acquired with the Aperio VERSA 8 (Leica) multifunctional scanner at 20×magnification. The intensities of DAB staining were measured and quantified with integrated optical density or cell intensity by Image Pro Plus 6 (Media Cybernetics).

#### 2.7.5 Bulk RNA sequencing

Upon completion of the experimental phase, gingival tissues surrounding the first molars were harvested and promptly homogenized in 2 mL of TRIzol reagent (Takara). Total RNA was isolated utilizing the RNeasy Kit (QIAGEN) following the manufacturer's protocol. The concentration and chemical purity of the extracted RNA were quantified via NanoDrop 2000 spectrophotometry (Thermo Fisher Scientific), while RNA integrity was rigorously validated using the Agilent 2100 Bioanalyzer with the RNA Nano 6000 Assay Kit, ensuring all samples met the quality threshold for downstream analysis.

To construct the sequencing libraries, 10 μg of high-quality total RNA was subjected to poly(A) mRNA enrichment using NEBNext Oligo d(T)25 Magnetic Beads. Subsequent cDNA synthesis and library preparation were executed using the NEBNext Ultra II Non-Directional and Vazyme TruePrep V2 kits, respectively. Sequencing was performed on the Illumina HiSeq X Ten platform, employing a 150-bp paired-end strategy.

Raw data underwent stringent quality control using FastQC (v0.11.9) and Trim Galore (v0.6.4_dev) to remove low-quality reads and adapter sequences with default parameters. The processed reads were aligned to the rat reference genome (Rattus_norvegicus, Ensembl 115) using Hisat2 (v2.0.5), permitting a maximum of two mismatches. Quantitation of gene expression was conducted through FeatureCounts (v2.0.0), with values normalized to FPKM. Differentially expressed genes (DEGs) between groups were identified using the DESeq2 package. The distribution and expression patterns of these DEGs were visualized using volcano plots and clustering heatmaps. Functional characterization was performed via Gene Ontology (GO) enrichment utilizing the clusterProfiler R package (v4.8.2) and MSigDB. Finally, track signals were visualized using the Integrative Genomics Viewer (IGV, v2.5.0).

### 2.8 Statistical analysis

Statistical analysis was performed with the aid of Origin software (version 2021), Graph prism software (version 8.0.2). All data are expressed as mean ± standard deviation, and at least 3 samples per experiment were used for statistical analysis of the data. Statistical analyses were performed by one-way analysis of variance (ANOVA) and Student's t-test, and p < 0.05 was considered statistically significant (∗p < 0.05, ∗∗p < 0.01, ∗∗∗p < 0.001, ∗∗∗∗p < 0.0001).

## 3 Results and discussion

### 3.1 Synthesis and characterization of NPs

PDA NPs were first synthesized via the self-oxidation of dopamine hydrochloride under mildly alkaline conditions. Simvastatin was subsequently loaded into the PDA NPs to obtain Sim@PDA NPs (Fig. 2A). The mass feed ratio of simvastatin to PDA NPs was optimized, and a final ratio of 1:1.5 (simvastatin toPDA NPs, w/w) was selected as it provided the optimal balance between drug loading and encapsulation efficiency (Supplementary Table S1). The morphology of the NPs was characterized via TEM and SEM. Both PDA and Sim@PDA NPs exhibited a uniform spherical morphology with consistent particle size. Following simvastatin loading, a slight yet negligible increase in average particle size was observed (Fig. 2B–E). Dynamic light scattering (DLS) analysis revealed that the hydrodynamic diameters of PDA NPs and Sim@PDA NPs were 199.27 ± 2.83 nm and 244.47 ± 3.44 nm, respectively (Fig. 2F and G).

**Fig. 2:**
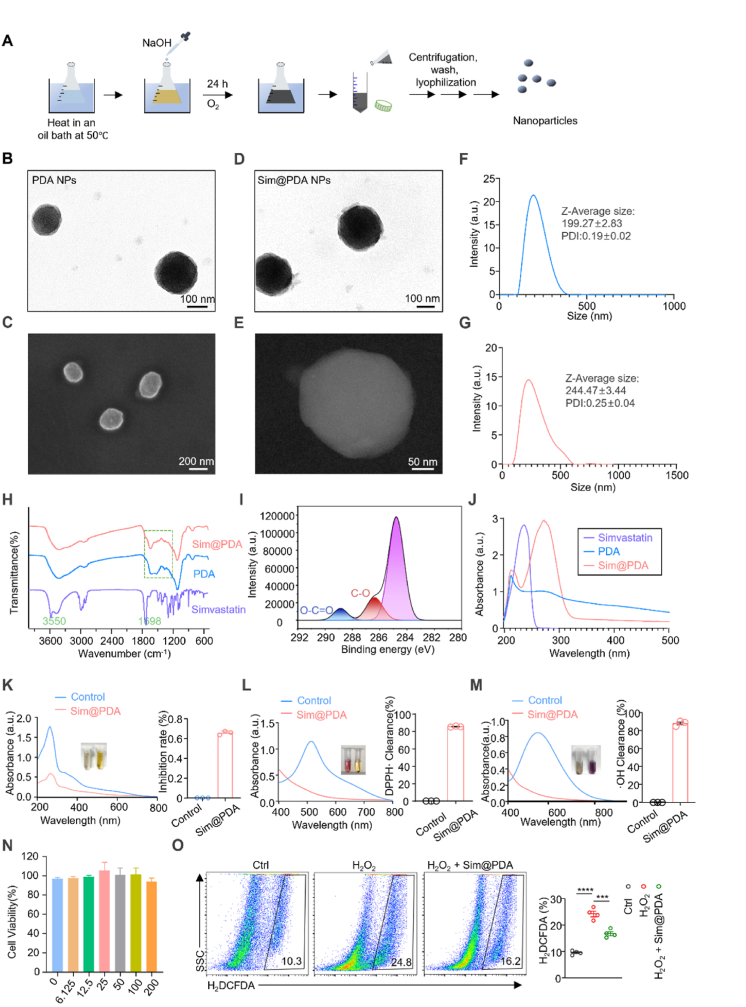
Synthesis and characterization of Sim@PDA nanoparticles. (A) Schematic illustration of the synthesis of Sim@PDA NPs. Representative (B) TEM and (C) SEM image of PDA NPs. Representative (D) TEM and (E) SEM image of Sim@PDA NPs. (F) DLS of PDA NPs. (G) DLS of Sim@PDA NPs. (H) FTIR spectra of Sim@PDA NPs, PDA NPs, simvastatin, respectively. (I) XPS C1s spectra of Sim@PDA NPs. (J) UV-vis spectra of simvastatin, PDA NPs, Sim@PDA NPs. (K) SOD-like enzyme activities of Sim@PDA NPs. Radical scavenging rate of Sim@PDA NPs toward (L) DPPH· and (M) ·OH. Each point represents mean ± standard deviation (SD) (n = 3). (N) Cell viability after treatment with different concentrations of NPs, as assessed by the CCK-8 assay. (O) Quantification of intracellular ROS levels via flow cytometry using a fluorescent probe.

To verify successful simvastatin incorporation, Fourier transform infrared (FTIR) spectroscopy was performed. Simvastatin exhibited characteristic peaks at 1698.59 cm^−1^ (lactone C=O stretching), 3550.73 cm^−1^ (O-H stretching), and multiple ester-related peaks in the 1300-1000 cm^−1^ region (Fig. 2H). In contrast, these characteristic absorption peaks were absent in the spectra of Sim@PDA NPs, accompanied by subtle spectral shifts relative to drug-free PDA NPs, likely due to hydrogen bond formation between the carbonyl groups of simvastatin and PDA NPs, indicating successful encapsulation of simvastatin within the carrier. Furthermore, X-ray photoelectron spectroscopy (XPS) was employed to probe the surface chemical states. The high-resolution C1s spectrum of Sim@PDA NPs (Fig. 2I) showed a significant increase in the proportion of C=O and C–O bonds compared to blank PDA NPs, which is directly attributable to the abundant carbonyl and ester groups present in the loaded simvastatin molecules, providing direct evidence of successful drug incorporation. Moreover, UV-vis spectroscopy revealed that Sim@PDA NPs displayed strong absorption peaks at 207 nm and 269 nm (Fig. 2J), corresponding to the characteristic signals of simvastatin and PDA NPs, further confirming successful encapsulation of Simvastatin within PDA NPs.

Beyond their function as a drug carrier, the polydopamine (PDA) matrix of the NPs possesses intrinsic antioxidant activity, which is crucial for modulating the chronic inflammatory microenvironment of bone defects [62]. The phenolic hydroxyl groups on PDA can effectively scavenge excessive reactive oxygen species (ROS), thereby alleviating oxidative stress and promoting a healing-favorable milieu [63]. The prolonged biodegradation property of PDA NPs [64] ensures the sustained antioxidant capability, working in concert with the early-phase drug release, constitutes a synergistic strategy for bone regeneration. Building on the drug release profile, we further investigated the antioxidant properties of Sim@PDA NPs, as mitigating oxidative stress is crucial for periodontal bone regeneration. The antioxidant capacity of Sim@PDA NPs was systematically evaluated using a series of *in vitro* assays, including pyrogallol autoxidation, DPPH radical scavenging, and hydroxyl radical (·OH) scavenging (salicylic acid-Fenton reaction) methods. The results demonstrated that Sim@PDA NPs possessed significant SOD-like enzyme activity, achieving scavenging rates of nearly 90% against both DPPH and ·OH radicals (Fig. 2K–M). Next, to ensure the biocompatibility prerequisite for any biomedical application, cytotoxicity was assessed. The CCK-8 assay revealed that cell viability remained above 95% even at the highest concentration (100 μg/mL), indicating excellent cytocompatibility (Fig. 2N). Subsequently, the ability of Sim@PDA NPs to reduce intracellular ROS was evaluated using the DCFH-DA fluorescent probe. As shown in Fig. 2O, the fluorescence intensity in the NPs-treated group was significantly lower than that in the H_2_O_2_-induced group, confirming their effective ROS scavenging function within a cellular context.

Collectively, PDA NPs function not only as efficient carriers for localized simvastatin delivery to periodontal tissues but also as intrinsic ROS scavengers, making them particularly suitable for the local treatment of diabetic periodontitis.

### 3.2 Preparation and characterization of MN patch

To achieve simultaneous inflammation control and periodontal tissue regeneration, we designed a core-shell MN patch that enables time-programmed release of doxycycline together with sustained release of simvastatin (hereafter termed as Doxy/SIM MNs). The MN patches were fabricated by sequentially depositing distinct materials into molds followed by centrifugal drying, yielding a well-defined core-shell structure (Fig. 3A). The fabricated MN patch consists of 5 × 10 MN array within the area of 0.25 cm^2^, with each MN 850 μm in height and 300 μm in base diameter. The MNs exhibited intact needle bodies with a conical morphology (Fig. 3B). To visualize the architecture, rhodamine B and rhodamine 123 were incorporated into the shell and core layers, respectively, demonstrating a clearly demarcated core-shell configuration under fluorescence imaging (Fig. 3C).

**Fig. 3:**
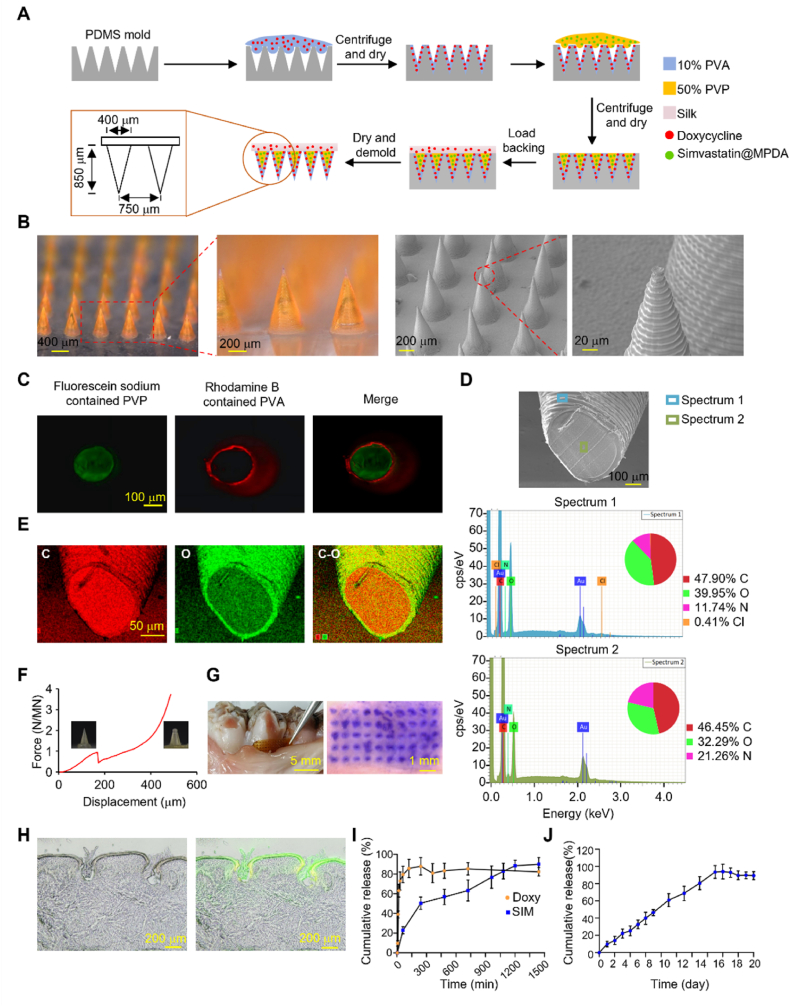
Fabrication and characterization of the Doxy/SIM MN patch. (A) Schematic illustration of the fabrication process of the Doxy/SIM MN patch. (B) Optical and SEM images of a fabricated MN patch. (C) Representative images of the core-shell structured MNs: Rhodamine B -labeled PVA shell (red) and Rhodamine 123 -labeled PVP core (green). (D) Cross-sectional SEM image of a single MN with blue and green rectangles indicating the shell and core layers, respectively, and corresponding EDS point analysis. (E) Elemental distribution maps of C and O on the cross-section of a single Doxy/SIM MN. (F) Force-displacement curve of the Doxy/SIM-MNs (inset: optical images of the MNs before and after the mechanical strength test). (G) Representative bright-field microscopy image of porcine gingival tissue after the application of the Doxy/SIM MN patch. (H) Histological analysis of the MN patch application site on porcine gingival tissue. (I) Cumulative release profiles of doxycycline (from the shell layer) and simvastatin (from the core) from the MN patch. Data are presented as mean ± SD (n = 4). (J) Cumulative release profile of doxycycline from the silk fibroin backing layer of the MN patch. Data are presented as mean ± SD (n = 4). (For interpretation of the references to colour in this figure legend, the reader is referred to the Web version of this article.)

Hydrophilic poly (vinyl alcohol) (PVA) was employed in the shell to encapsulate doxycycline for rapid release, while hydrophobic poly (vinylpyrrolidone) (PVP) was used in the core to encapsulate Sim@PDA NPs. Crucially, the core also imparts essential mechanical robustness to the microneedle. This is further corroborated by the data in Fig. S2A–C, which demonstrates that a microneedle fabricated entirely from PVA fails to penetrate *ex vivo* gingival tissue (Fig. S2A and B)and exhibits markedly lower mechanical strength (<0.02 N per needle) (Fig. S2C), whereas the core-shell configuration successfully achieves penetration, highlighting the necessity of the core-shell design for structural integrity. Moreover, a deliberate strategy was employed in fabricating the outer and core layers using aqueous and ethanol-based systems, respectively. This approach effectively prevents the diffusion of doxycycline from the outer layer into the core, owing to the insolubility of the outer PVA in ethanol. Elemental energy-dispersive X-ray spectroscopy (EDS) and mapping confirmed the compositional distinction, the shell displayed higher oxygen content consistent with PVA (Fig. 3D), while chlorine signals—attributable to doxycycline—were detected exclusively in the shell, confirming its selective encapsulation as designed (Fig. 3E).

Mechanical strength analysis using a digital force analyzer showed that each PVA/PVP composite microneedle could withstand a force exceeding 0.92 N (Fig. 3F). Ex vivo penetration into porcine gingiva was confirmed by crystal violet staining of puncture sites (Fig. 3G) and cryosectional imaging of inserted microneedles (Fig. 3H), verifying sufficient robustness for mucosal application. Furthermore, the MN patches exhibited satisfactory storage stability, maintaining their morphological integrity and mechanical strength (with variations of less than 0.3 N) for at least 4 weeks under ambient, dry conditions (Fig. S3).

Next, the drug release kinetics of the microneedle patch were examined by UV-vis spectroscopy. As shown in Fig. 3I, doxycycline loaded in the PVA shell exhibited a rapid burst release, with ∼39% of the drug released within the first 10 min and cumulative release reaching ∼85% within 2 h (yellow circles). This is designed to ensure the immediate establishment of a bactericidal concentration in the periodontal pocket. Concurrently, simvastatin released from the PVP core showed a release profile with over 80% of the drug released within 24 h (Fig. 3I, blue squares). Delivering an osteogenic signal while the antioxidant polydopamine carrier mitigates local oxidative stress. In contrast, the release from the silk fibroin backing layer followed a sustained pattern, with doxycycline release detectable for up to 16 days (Fig. 3J), aiming to suppress pathogen recolonization. Collectively, the sequential release profiles from the shell (instant antimicrobial), core (osteogenic), and backing (prolonged antimicrobial) are designed to meet the therapeutic challenge of coordinating antimicrobial and regenerative phases, which is difficult to achieve with single-release-profile systems. In comparison with other advanced local delivery systems for periodontitis—such as injectable hydrogels, in-situ forming films, or nanoparticle suspensions—our core-shell MN patch offers distinct practical advantages. A review of the literature indicates that while hydrogels and films can provide sustained release, they may face challenges regarding reliable retention within the dynamic periodontal pocket [7]. In contrast, MN system provides mechanical self-anchorage, ensuring prolonged residence at the disease site and enabling direct intra-tissue drug delivery across the sulcular epithelium, which facilitates higher local bioavailability. Moreover, the core-shell design permits precise spatiotemporal control over multiple drugs, a feature not easily accomplished with single-matrix systems.

### 3.3 Evaluation of the antibacterial properties of MNs

Dental plaque biofilm is the primary etiological factor in periodontitis. Within the subgingival plaque, *Porphyromonas gingivalis* (*P. gingivalis*) is the pathogen most strongly and consistently associated with chronic periodontitis [[65], [66], [67]]. Therefore, the antibacterial function of microneedle patches - particularly their efficacy against *P. gingivalis* - is crucial for inhibiting disease progression. The antibacterial efficacy of MNs was first evaluated *in vitro* using plate counting assays. After 48 h co-incubation of *P. gingivalis* with MN extracts, plating revealed markedly fewer colonies in the Doxy-MNs, Doxy/SIM-MNs, and Periostat® (commercial minocycline gel, which was considered a benchmark therapy in clinical periodontitis treatment [68]) groups compared with the Control (Fig. S4A). As expected, Blank-MNs and SIM-MNs did not show obvious antibacterial effects. Similarly, when co-cultured with *Staphylococcus aureus* (*S. aureus*) for 24 h, Doxy-MNs and Doxy/SIM-MNs again suppressed bacterial growth, with effects comparable to Periostat®.

Further antibacterial activity was confirmed via inhibition zone assays. As shown in Fig. 4A–D, both Doxy-MNs and Doxy/SIM-MNs generated clear inhibition zones against both *S. aureus* and *P. gingivalis*, with diameters of 31.92 ± 0.74 mm and 82.17 ± 5.99 mm for Doxy-MNs, and 30.19 ± 1.75 mm and 79.15 ± 9.73 mm for Doxy/SIM-MNs, respectively, which were comparable to Periostat®. By contrast, Control and Blank-MNs groups displayed negligible inhibition zones. These results demonstrate that Doxy-loaded MNs exert potent antibacterial effects, particularly against the anaerobe *P. gingivalis*. Live/dead bacterial staining assays further validated these findings (Fig. 4E and F). As shown in Fig. 4I, Doxy-MNs, Doxy/SIM-MNs, and Periostat® treatment markedly reduced bacterial viability, with extensive bacterial death compared with other groups. SEM imaging provided additional mechanistic insights: bacteria treated with Doxy-MNs or Doxy/SIM-MNs exhibited disrupted cell walls and extensive leakage of intracellular contents, in sharp contrast to the intact morphologies observed in Control and Blank-MNs groups (Fig. 4E and F). These findings suggest that Doxy-loaded MNs induce bacterial death primarily through cell membrane disruption.

**Fig. 4:**
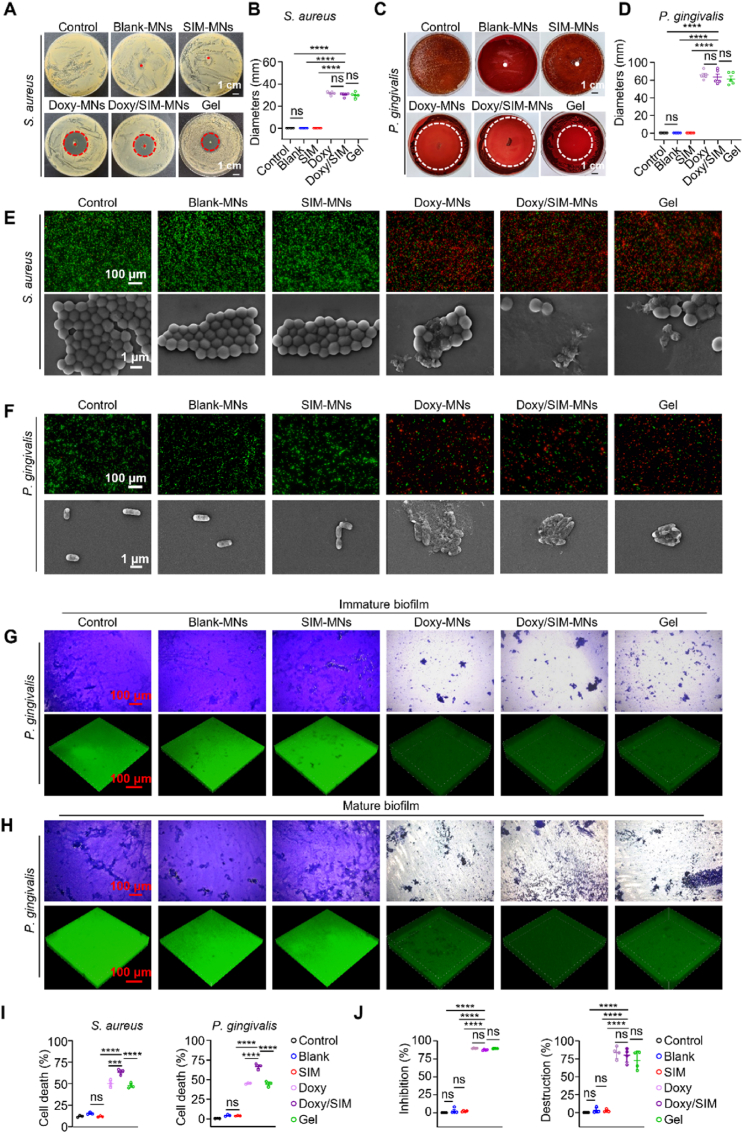
*In vitro* antibacterial performance of Doxy/SIM-MNs. (A, C) Representative photographic images of the inhibition zones against (A) *S. aureus* and (C) *P. gingivalis* after different treatments. The bolded dots indicate the application sites of the MN patches, and the circles outline the inhibition zones. (B, D) Corresponding quantitative analysis of the inhibition zones against (B) *S. aureus* and (D) *P. gingivalis* (n = 5). (E, F) Live/dead staining images and FESEM images of (E) *S. aureus* and (F) *P. gingivalis* after different treatments. (G, H) Antibiofilm activity against (G) immature and (H) mature *P. gingivalis* biofilms, showing photographs of crystal violet-stained biofilms and confocal 3D images of SYTO-9-stained biofilms. (I) Quantitative analysis of bacterial viability based on Live/Dead staining (n = 4). (J) Crystal violet quantification of biofilm biomass, showing inhibition and destruction rates for immature and mature biofilms, respectively (n = 4). Data are presented as mean ± SD. ∗*P* < 0.05, ∗∗*P* < 0.01, ∗∗∗*P* < 0.001, ∗∗∗∗*P* < 0.0001; ns indicates no significance. (For interpretation of the references to colour in this figure legend, the reader is referred to the Web version of this article.)

Bacterial biofilms-composed of polysaccharides, fibrin, lipoproteins, and other extracellular polymers that greatly hinder conventional drug penetration and efficacy [69]. MNs represent a promising strategy to combat biofilm-associated infections: they can physically breach biofilm barriers to deliver antibiotics directly, while their material-specific properties provide sustained antimicrobial activity [68]. As shown in Fig. 4G, Doxy-MNs, Doxy/SIM-MNs, and Periostat® markedly suppressed *P. gingivalis* biofilm formation, with inhibition rates of 89.90 ± 0.35%, 87.59 ± 0.81%, and 89.48 ± 0.14%, respectively, as confirmed by crystal violet staining and confocal microscopy (Fig. 4J). Moreover, when applied to pre-formed mature biofilms, Doxy-MNs and Doxy/SIM-MNs significantly disrupted biofilm structure: crystal violet staining revealed reduced biomass, while confocal 3D imaging showed thinner biofilm layers with weakened green fluorescence (Fig. 4H). Quantitative analysis indicated biofilm destruction rates of 83.68% and 79.90% for Doxy-MNs and Doxy/SIM-MNs, respectively, comparable to Periostat® (Fig. 4J). Collectively, these findings highlight the superior antibacterial and antibiofilm efficacy of doxycycline-loaded MNs, especially against anaerobic periodontal pathogens, thereby providing a promising strategy for the management of biofilm-associated periodontitis.

### 3.4 Biosafety evaluation of doxy/SIM-MNs *In Vitro*

Cytocompatibility represents a fundamental prerequisite for the *in vivo* application of MN patches. To this end, a hemolysis assay was first performed. Red blood cells treated with MN extracts maintained intact morphology, and the hemolysis ratio remained below 5%, with no obvious hemolytic effect observed (Fig. S5B), indicating the favorable cytocompatibility of the MN patches. Furthermore, as shown in Fig. 5A and S5A, after 24 h of co-culture C3H10T1/2 or NIH-3T3 cells with different MNs extracts, both cells under different MN extracts treatment exhibited comparable green fluorescence signals relative to the control group, with only negligible red fluorescence detected, further validating the favorable compatibility of the MN patches. Collectively, these results confirm that the Doxy/SIM-MNs possess excellent biosafety and hold substantial promise for clinical translation.

**Fig. 5:**
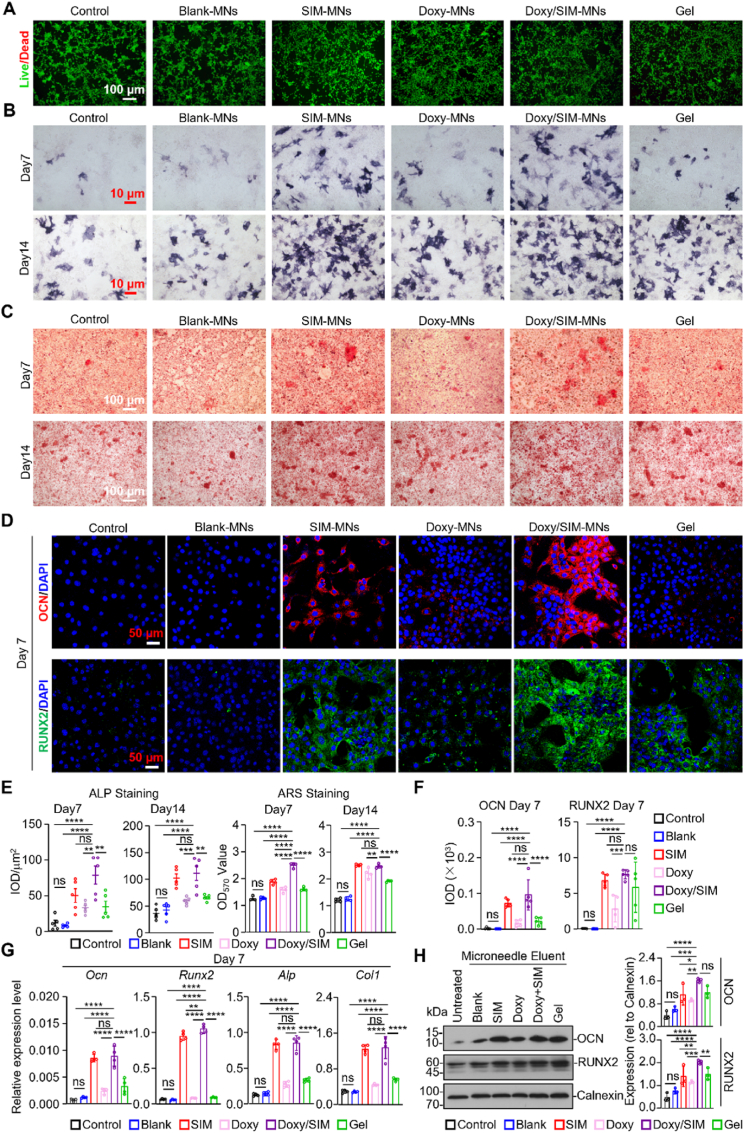
Cellular biocompatibility and osteogenic properties of the Doxy/SIM MN patch. (A) Live/Dead staining of C3H10T1/2 cells co-cultured with different samples for 24 h. (B, C) (B) ALP and (C) Alizarin Red S (ARS) staining of C3H10T1/2 cells cultured with different samples for 7 and 14 days. (D) Representative immunofluorescence images of OCN and RUNX2 in C3H10T1/2 cells cultured with different samples for 7 days. (E, F) Quantitative analyses of the results presented in panels B-D. (E) Quantitative assessment of ALP and ARS staining shown in (B, C) (*n* = 5). (F) Statistical analysis of the mean fluorescence intensity (MFI) of OCN and RUNX2 from (D) (n = 5). (G) Relative mRNA expression of *Runx2, Alp, Col1,* and *Ocn* in C3H10T1/2 cells treated with different samples for 7 days (n = 4). (H) Western blot analysis and corresponding quantification of osteogenesis-related protein (OCN and RUNX2) in C3H10T1/2 cells after different treatments for 7 days. Data are presented as mean ± SD. ∗*P* < 0.05, ∗∗*P* < 0.01, ∗∗∗*P* < 0.001, ∗∗∗∗*P* < 0.0001; ns indicates no significance. (For interpretation of the references to colour in this figure legend, the reader is referred to the Web version of this article.)

### 3.5 Evaluation of the osteogenic differentiation potential of doxy/SIM-MNs *In Vitro*

C3H10T1/2 cells are considered to be at an early stage of mesenchymal lineage commitment, and therefore have been widely employed as a model system for both stem cell biology and osteogenic/chondrogenic differentiation. After confirming the cytocompatibility of the MN patches *in vitro*, we next evaluated their osteogenic potential. C3H10T1/2 cells were subjected to osteogenic induction for 7 and 14 days, followed by ALP staining (Fig. 5B). On day 7, except for the Control and Blank-MN groups, all other groups exhibited appreciable ALP deposition (Fig. 5E). By day 14, ALP activity was markedly increased across all groups, with the Doxy/SIM-MNs showing the highest ALP levels at both time points (Fig. 5E).

To investigate calcium deposition after MNs treatment, Alizarin Red S (ARS) staining was performed on C3H10T1/2 cells after 7 and 14 days of osteogenic induction (Fig. 5C). At both time points, the Doxy/SIM-MNs group exhibited the highest density of calcium nodules, followed by the SIM-MNs group, whereas Doxy-MNs and Gel groups showed moderate increases compared with the Control group. In contrast, the Blank-MNs group displayed the fewest calcium nodules. Quantitative analysis of calcium content (Fig. 5E), using 10% (w/v) cetylpyridinium chloride extraction, confirmed that the Doxy/SIM-MNs group achieved the highest calcium deposition, indicating the most robust mineralization, whereas the Blank-MNs group showed minimal calcium levels, comparable to the Control.

After 7 days of osteogenic induction, immunofluorescence staining for RUNX2 and OCN was performed (Fig. 5D). Fluorescence intensity analysis revealed trends consistent with ALP and ARS staining results (Fig. 5F). Specifically, the Doxy/SIM-MNs group displayed the strongest fluorescence signals, significantly higher than those observed in the SIM-MNs, Doxy-MNs, and Gel groups. In contrast, the Blank-MNs group exhibited fluorescence comparable to the Control, with weak expression of osteogenic markers. These results demonstrate an enhancement in the protein expression of osteogenic markers following Doxy/SIM-MNs treatment.

To further validate the osteogenic effect at the gene level, RT-qPCR was employed to measure the mRNA expression of *Alp*, *Runx2*, *Col1*, and *Ocn* (Fig. 5G). *RUNX2* is a pivotal transcription factor for osteoblast differentiation, critically regulating the expression of bone-specific genes [70]. *Col1* is a major component of the bone extracellular matrix, facilitating osteoblast adhesion and differentiation [71]. *Ocn* is a late-stage osteoblast marker, specifically expressed in osteogenic cells, and regulates calcium homeostasis and mineralization through calcium binding [72]. Compared with the Control group, Doxy/SIM-MNs and SIM-MNs significantly upregulated all four osteogenic markers. Consistent trends were observed at the protein level for RUNX2 and OCN, as revealed by Western blot analysis (Fig. 5H).

Collectively, these results demonstrate that Doxy/SIM-MNs markedly promote osteogenic differentiation of multipotent mesenchymal cells *in vitro*. This was evidenced by increased ALP activity, enhanced calcium deposition, and elevated mRNA and protein expression of osteogenic markers across multiple assays, highlighting the potent osteoinductive capability of Doxy/SIM-MNs. These findings are consistent with previous reports of simvastatin-mediated enhancement of osteogenic differentiation.

### 3.6 Successful establishment of a diabetic periodontitis rat model

Diabetic periodontitis rats exhibited typical diabetes-related clinical manifestations, including polydipsia, polyphagia, polyuria, dull fur, abnormal odor, and a hunched posture. The successful establishment of the type 2 diabetes model was confirmed by an oral glucose tolerance test (OGTT). As shown in Fig. S7A, blood glucose levels in both diabetic and healthy groups rose within 30 min after oral glucose administration and subsequently declined. At 2 h post-administration, blood glucose in the healthy control group returned to normal, whereas diabetic rats maintained levels above 20.13 mmol/L, confirming the successful induction of diabetes. On this basis, periodontitis was induced by placing ligature wires around the bilateral maxillary first molars. At the end of the experimental period, removal of the ligatures revealed substantial accumulation of dental plaque and food debris around the cervical region of the first molars, pronounced gingival redness and swelling, increased bleeding on probing, and noticeable tooth mobility. Micro-CT 3D reconstruction of the maxillae (Fig. 6B) demonstrated significant interproximal spacing between the first and second molars, reduced alveolar bone height, root exposure, and apparent bone defects in the furcation area in the Control group, indicating successful establishment of the diabetic periodontitis model.

**Fig. 6:**
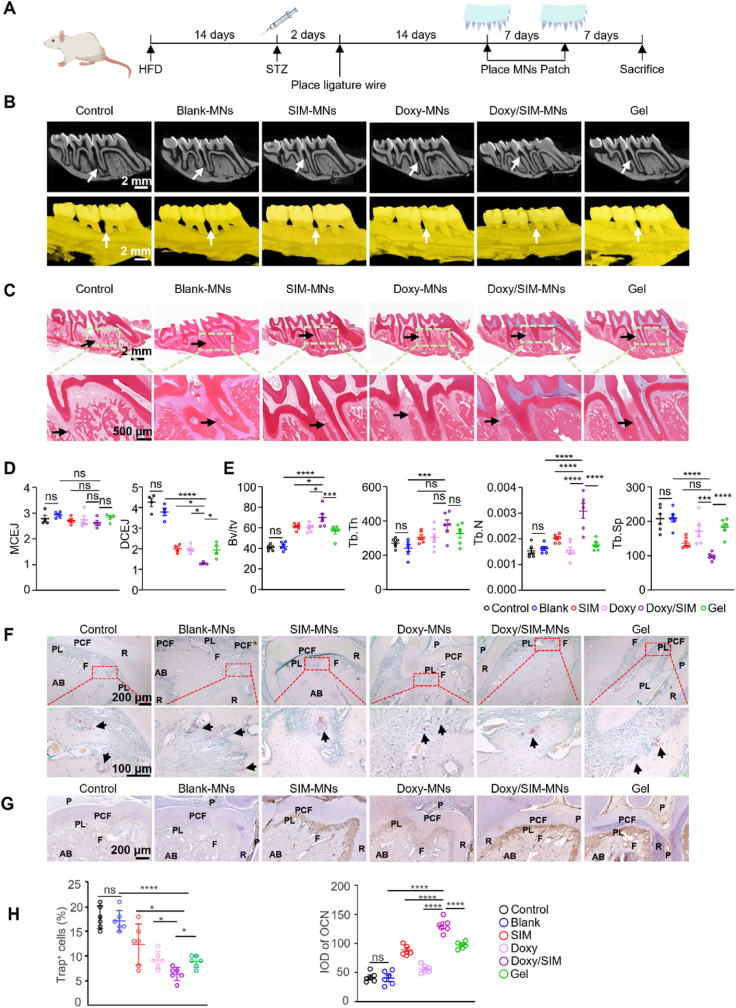
*In vivo* evaluation of the therapeutic effect of the Doxy/SIM MN patch on periodontitis with diabetes. (A) Schematic diagram of the animal experiment. (B) Representative micro-CT images of the alveolar bone. (C) Representative hematoxylin and eosin (H&E) staining of the periodontal tissue.(D) The measurement of cementoenamel junction to alveolar bone crest (CEJ-ABC) distance at both the mesial and distal aspects of the maxillary first molar in rats (n = 4). (E) Quantitative bone mass analysis from micro-CT data (n = 6). (F) Representative tartrate-resistant acid phosphatase (TRAP)-stained sections of the periodontium. (Key anatomical structures are labeled as: R, root; PL, periodontal ligament; P, dental pulp; F, furcation area; AB, alveolar bone; PCF, pulp chamber floor. Black arrows indicate TRAP-positive cells.) (G) Representative immunohistochemical (IHC) staining for osteocalcin (OCN) in the periodontal tissue. (Key anatomical structures are labeled as: R, root; PL, periodontal ligament; P, dental pulp; F, furcation area; AB, alveolar bone; PCF, pulp chamber floor.) (H) Quantitative analysis of the number of osteoclasts per field from TRAP staining and the OCN expression level from IHC staining (n = 6). Data are presented as mean ± SD. ∗*P* < 0.05, ∗∗*P* < 0.01, ∗∗∗*P* < 0.001, ∗∗∗∗*P* < 0.0001; ns indicates no significance.

### 3.7 Therapeutic effects of doxy/SIM-MNs on diabetic periodontitis in rats

To evaluate the *in vivo* therapeutic efficacy of the microneedle patch, a rat model of periodontitis complicated by diabetes was utilized. On day 14, the animals were subsequently allocated into six groups receiving the following treatments: 1) Control (no treatment), 2) Blank-MNs (blank MN patches without drug loading), 3) Doxy-MNs (MN patches with doxycycline-loaded shell), 4) SIM-MNs (MN patches with Sim@PDA-MNs (MN patches with simvastatin-loaded core only), 5) Doxy/SIM-MNs (MN patches co-loaded with doxycycline and simvastatin), and 6) Gel (minocycline hydrochloride gel) (Fig. 6A). Micro-CT 3D imaging revealed that the Blank-MNs and untreated Control groups exhibited comparable reductions in alveolar bone height, with distal alveolar bone resorption extending to near the root apex on sagittal planes. Intervention with SIM-MNs, Doxy-MNs, or Gel partially alleviated alveolar bone resorption, whereas the Doxy/SIM-MNs group exhibited the minimal resorption, less than one-third of the root length (Fig. 6B). Similar trends were observed in H&E-stained sections, which also showed concomitant loss of periodontal soft tissues along with alveolar bone resorption. Quantitative measurement of the cementoenamel junction (CEJ) to alveolar bone crest (ABC) distances for the mesial and distal regions of the first maxillary molars (Fig. 6D) revealed significant differences in distal CEJ-ABC distances among treatment groups compared with untreated controls, whereas mesial CEJ-ABC distances showed no significant changes. This may be attributed to more efficient self-cleansing of the mesial region, resulting in less plaque accumulation compared with the distal interproximal region, as well as the influence of sagittal imaging angles. Analysis of distal CEJ-ABC distances and related bone density metrics clearly reflected post-treatment alveolar bone changes. Compared with Control and Blank groups, the Doxy/SIM-MNs group showed significantly reduced CEJ-ABC distances (p < 0.05). Trabecular thickness (Tb.Th) and trabecular number (Tb.N) were markedly increased, and bone volume fraction (BV/TV), the most direct indicator of bone density and quantity, also showed significant elevation, demonstrating that Doxy/SIM-MNs not only suppressed alveolar bone resorption in diabetic periodontitis rats but also improved bone quality, outperforming treatment with either doxycycline or simvastatin alone (Fig. 6E). To assess the anti-infective impact in the challenging oral microenvironment, plaque samples from the treatment sites were cultured. A visible reduction in bacterial growth was observed in the Doxy/SIM-MNs, Doxy-MNs, and Gel groups compared to the non- or sham-treated controls (Supplementary Fig. S8), corroborating the local release and activity of doxycycline *in vivo*. Building upon this foundational antimicrobial effect, TRAP staining and OCN immunohistochemistry confirmed that Doxy/SIM-MNs uniquely enhanced osteogenic activity (Fig. 6F–H), underscoring the added value of the combined simvastatin delivery.

To further investigate the underlying mechanisms by which the microneedle patches containing Simvastatin and doxycycline ameliorate diabetic periodontitis. We performed RNA sequencing analysis on the gingival soft tissues harvested from the Simvastatin plus doxycycline-treated (Dox/Sim) and Control (Ctrl) experimental rats. The transcriptomic profiling revealed a distinct set of differentially expressed genes (DEGs) between Dox/Sim-treated and control groups, with osteogenic markers (e.g., *Ibsp, Mgp,* and *Col11a2*) were markedly upregulated, whereas, inflammatory mediators (e.g., *Cxcl1, Fosl1,* and *Spp1*) were downregulated (Fig. 7A). GO enrichment analysis further demonstrated that Dox/Sim-treated groups were predominantly enriched for genes associated with ossification, biomineralization, and connective tissue development, whereas Ctrl enriched for genes associated with pro-inflammatory and apoptotic processes (Fig. 7B). Furthermore, signaling pathway enrichment analysis showed that simvastatin and doxycycline treatment relieved negative regulation of Wnt signaling, avtivation osteoclast differentiation and tissue regeneration programs, while suppressing NF-κB signaling and apoptosis (Fig. 7B). Genes expression heatmap and genome browser tracks confirmed enhanced transcriptional activity of representative osteogenic regulators (*Spp1, Ibsp, Gpc3,* and *Col11a2*), down-regulation of negative regulator of Wnt signaling (*Nkd1*, *Nkd2*, and *Dkk2*) and apoptosis (*Atf3*) in the Dox/Sim-treated group (Fig. 7D). At the protein level, immunoblotting demonstrated decreased levels of p65 phosphorylation (p-p65) and cleaved caspase-3, together with increased Lgr5 and E-cadherin expression, which were further quantitatively validated by densitometry analysis (Fig. 7E–F). Collectively, these results indicate that Simvastatin combined with doxycycline treatment fosters a pro-regenerative transcriptional landscape and enhances osteogenic potential, while mitigating inflammation and apoptosis in periodontal tissues. While these multi-omics data strongly implicate the modulation of Wnt and NF-κB signaling axes in the therapeutic outcome, future studies employing specific pathway modulators or genetic models are warranted to definitively establish causality within this complex network. Moreover, advancing this platform toward clinical translation will require further investigation into its long-term pharmacokinetics, optimal dosing, and safety profile in more translational animal models.

**Fig. 7:**
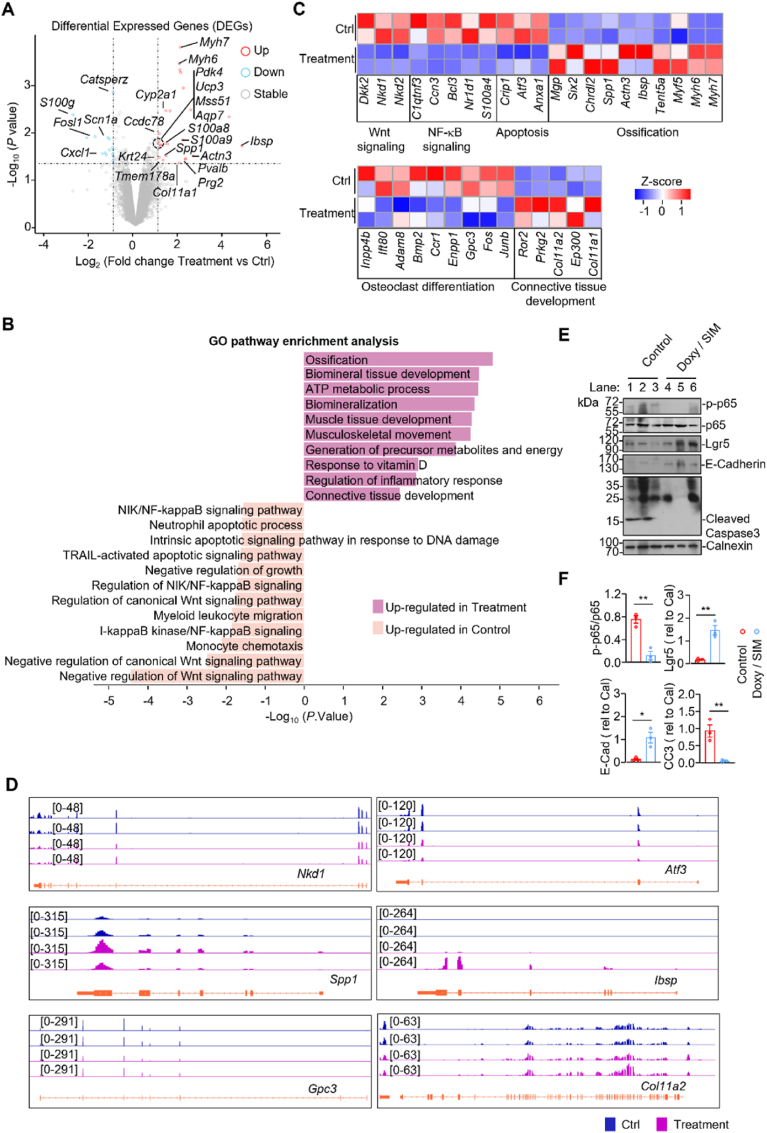
Transcriptional analysis elucidates the molecular mechanisms underlying the response to the Doxy/SIM MN patch treatment. (A) Volcano plot identifying significantly differentially expressed genes following treatment. Key upregulated and downregulated genes are highlighted. (B) Gene Ontology (GO) enrichment analysis of biological processes reveals treatment-induced alterations in ossification, Wnt/NF-κB signaling, and apoptosis. (C) Heatmap visualization corroborates the concerted dysregulation of genes within critical signaling pathways and osteochondral processes. (D) Genome browser tracks demonstrating treatment-specific alterations in chromatin accessibility at key gene loci (e.g., Nkd1, *Atrf*, *Spp1*, *Ibsp*, *Gpc3*, *Col11a2*), suggesting epigenetic regulation. (E, F) (E) Western blot analysis and (F) corresponding densitometric quantification of NF-κB signaling (increased p-p65/p65 ratio) and E-cadherin expression, validating transcriptomic findings at the protein level (n = 4). Data are presented as mean ± SD. ∗*P* < 0.05, ∗∗*P* < 0.01, ∗∗∗*P* < 0.001, ∗∗∗∗*P* < 0.0001; ns indicates no significance.

## 4 Conclusion

The treatment of periodontitis comorbid with diabetes is particularly challenging due to the intertwined issues of infection and impaired regeneration. To address this, we developed a core-shell MN patch designed for temporally controlled drug release to achieve synergistic therapy. The system first utilizes the rapid burst release of doxycycline for immediate biofilm clearance, while its backing layer enables sustained, long-term release to continuously inhibit pathogen recolonization, thereby effectively controlling infection and inflammation. Building upon this foundation, simvastatin delivered by polydopamine nanoparticles concurrently exerts dual effects of mitigating oxidative stress and promoting osteogenesis. This “anti-infection first, pro-regeneration next” strategy demonstrated synergistic efficacy in a diabetic rat periodontitis model: it significantly controlled infection while effectively promoting alveolar bone regeneration. This study provides a novel local delivery strategy featuring temporally programmed release for the treatment of diabetic periodontitis, integrating both anti-infective and bone-regenerative functions.

## CRediT authorship contribution statement

**Shengdan Zhang:** Conceptualization, Formal analysis, Investigation, Methodology, Validation, Writing – original draft. **Wen Zhang:** Conceptualization, Formal analysis, Investigation, Methodology, Writing – original draft. **You Wang:** Formal analysis, Investigation, Methodology, Validation, Writing – original draft. **Miao Yin:** Funding acquisition, Methodology, Validation. **Shuai Lu:** Funding acquisition, Project administration, Writing – review & editing. **Wei Li:** Conceptualization, Project administration, Supervision, Writing – review & editing. **Bo Cheng:** Conceptualization, Funding acquisition, Project administration, Supervision, Writing – review & editing.

## Declaration of competing interest

The authors declare that they have no known competing financial interests or personal relationships that could have appeared to influence the work reported in this paper.

## Figures and Tables

**figs1:**
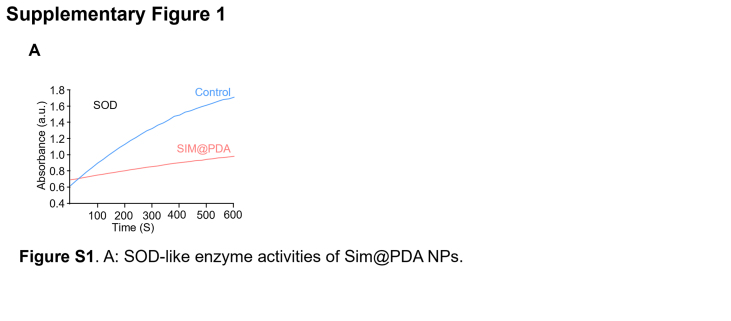


**figs2:**
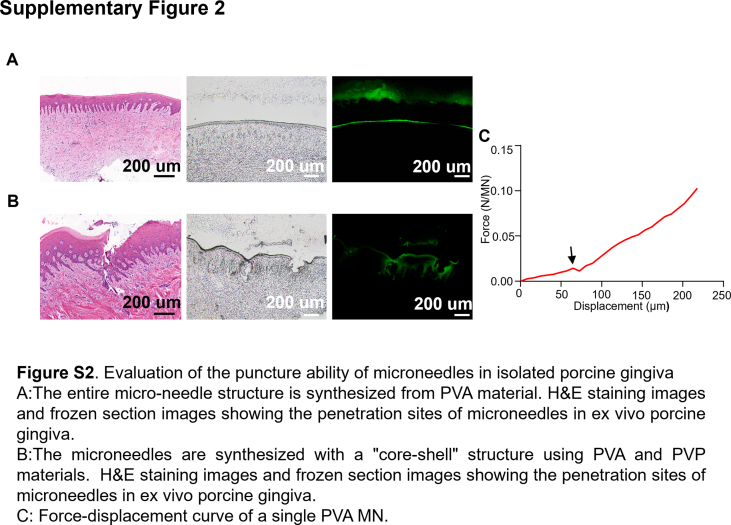


**figs3:**
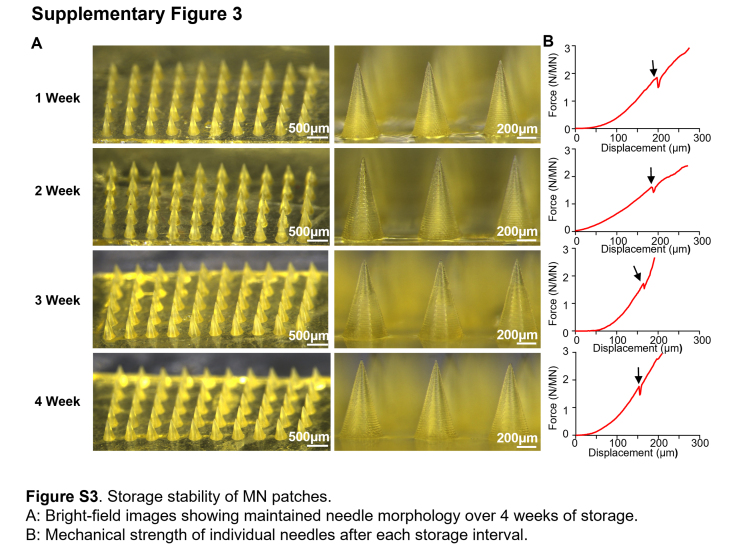


**figs4:**
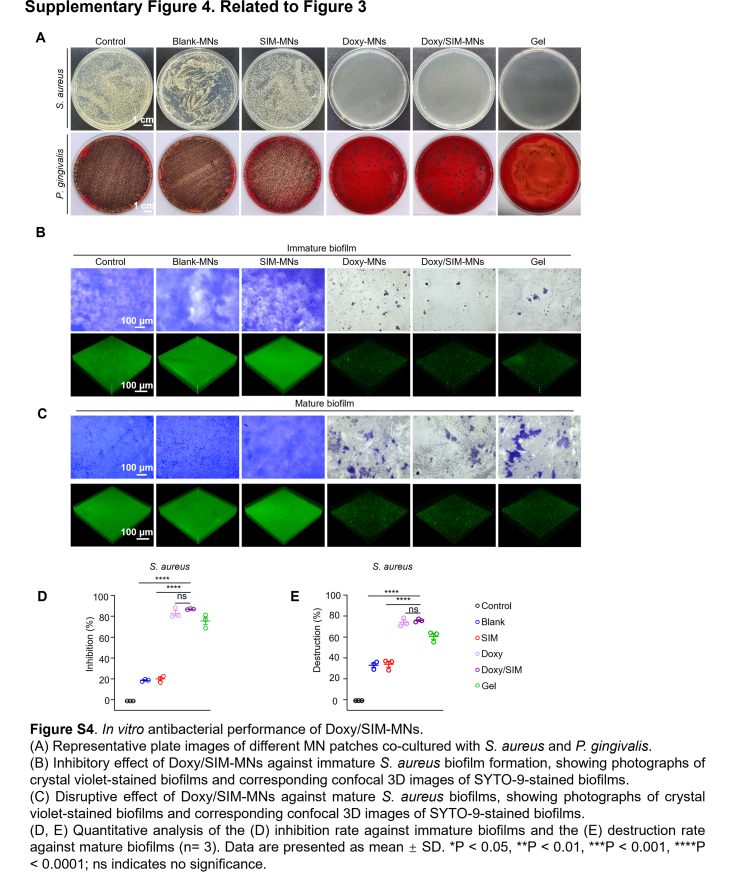


**figs5:**
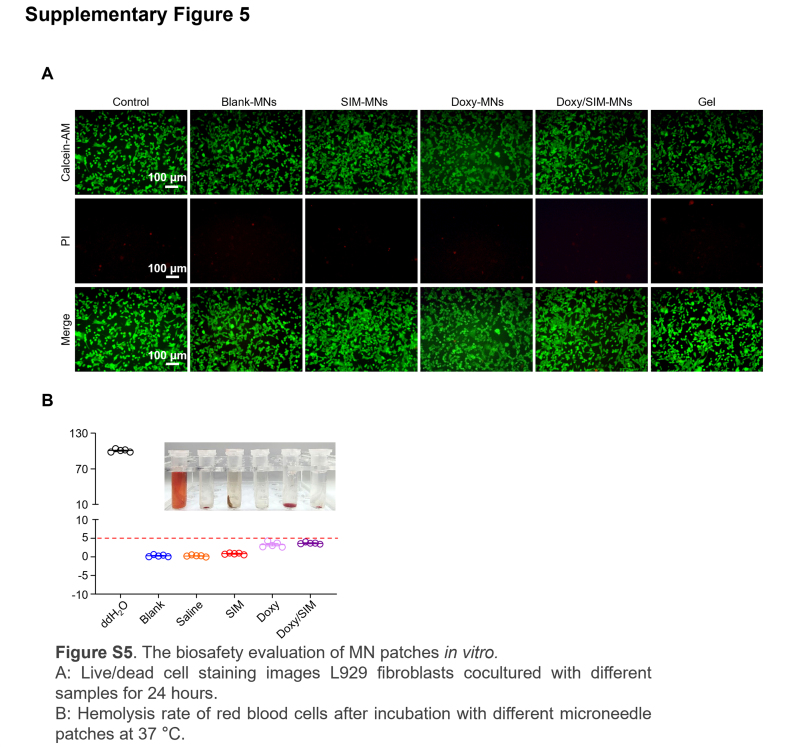


**figs6:**
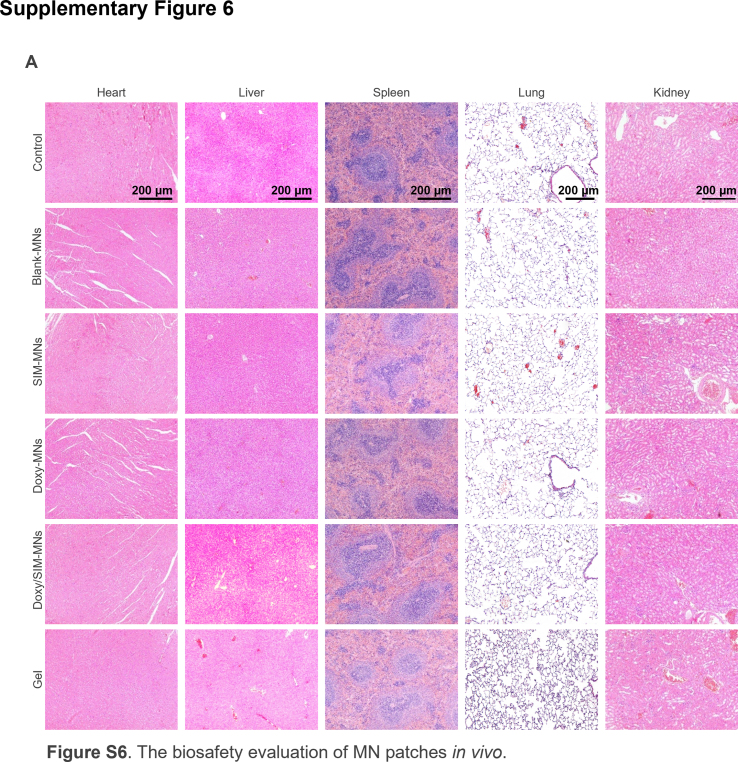


**figs7:**
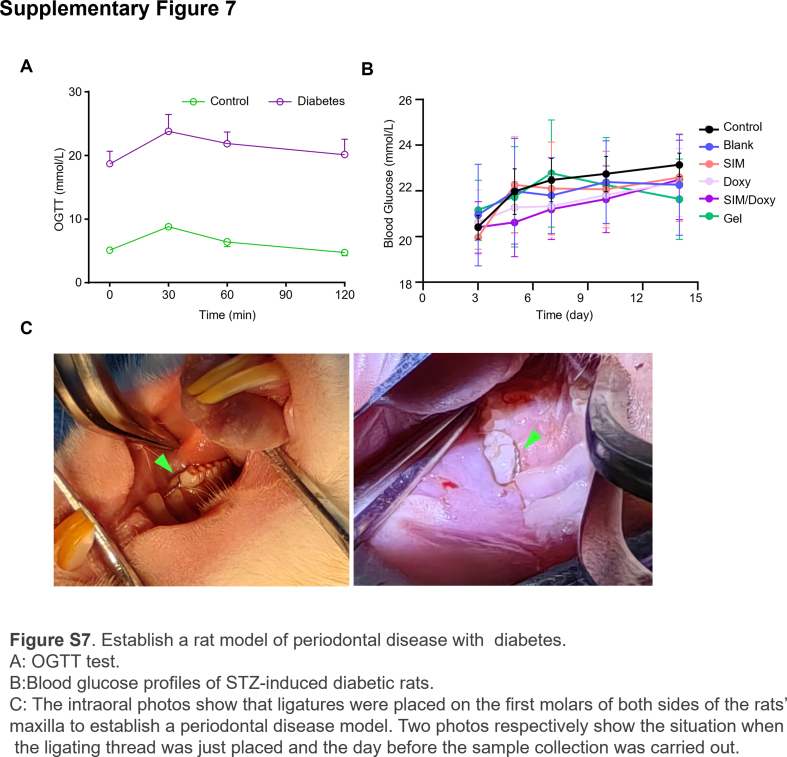


**figs8:**
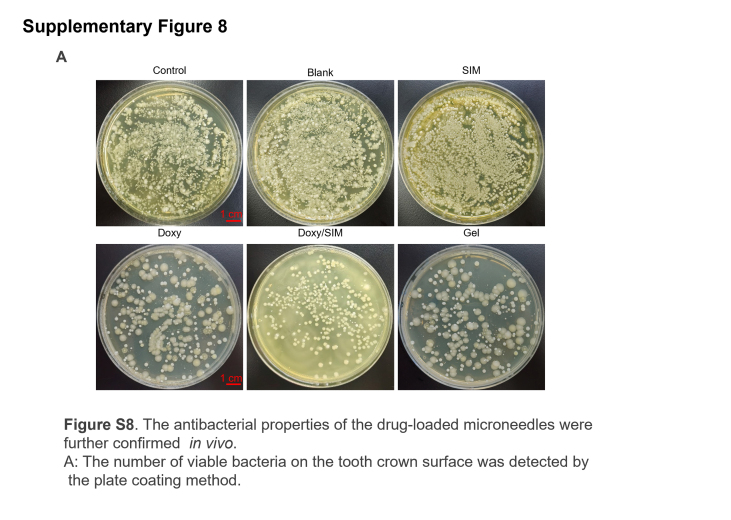


**figs9:**
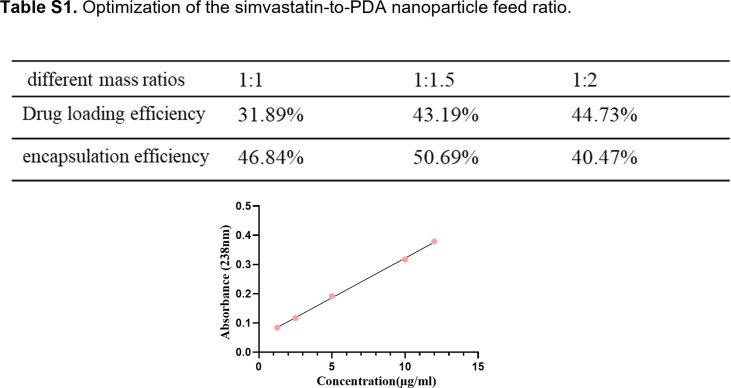


**figs10:**
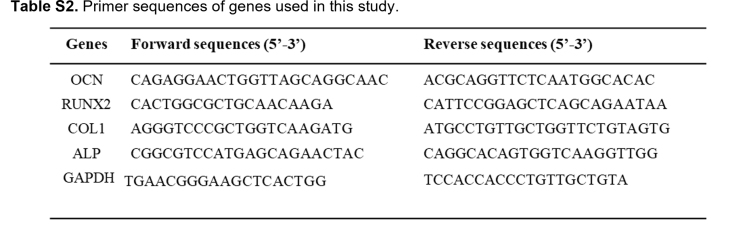


## Data Availability

Data will be made available on request.
